# Supplementary material for: Prenatal and Postpartum Maternal Iodide Intake from Diet and Supplements, Urinary Iodine and Thyroid Hormone Concentrations in a Region of the United Kingdom with Mild-to-Moderate Iodine Deficiency
Source: Nutrients. 2021 Jan 14;13(1):230. doi: 10.3390/nu13010230 (PMC7830764; doi:10.3390/nu13010230)
Supplement: Supplementary file 1 [file nutrients-13-00230-s001.pdf]

SUPPLEMENTARY MATERIAL

Table S1: Category details for dietary iodide source

Category name	Details
Dairy only	Foods where dairy is the only or major ingredient: Milk, cream, butter, cheese, yoghurt etc.
Dairy with cereal, with or without egg	Composite foods with a dairy and cereal content, with or without eggs. Including pizza, pasta dishes with dairy, pastry, biscuits, batters/pancakes, cakes/sponge, desserts, rice pudding, cheese sandwiches, custard, quiche etc.
Eggs, egg dishes	All eggs, egg curry and flan where eggs formed the main dish component and the dish contained little/no cereal or dairy
Cereals	Breads, rice and other grains or dishes prepared without a large proportion of dairy, fish, egg or meat
White fish	Haddock, cod etc. including battered and bread-crumbed fish
Other seafood	All seafood not classed as white fish, including salmon, trout, prawns etc.
Poultry or red meat and dishes	Poultry or meat as the only or main component of a dish
Fruit, vegetables, pulses and dishes	All raw or cooked fruit, vegetables, or pulses or dishes containing these without a large proportion of dairy, fish, egg or meat
Confectionery	Chocolate, filled chocolates and chocolate-coated biscuits/wafers
Other	Any other item not categorised above, including condiments, nuts and soy products

Table S2: Maternal characteristics according to use of iodide supplements in pregnancy

	All	No reported use of iodide containing supplements in pregnancy	Used iodide-containing supplement at any time in pregnancy
N	246	86	160
Age (years) Median (IQR)	31 (27, 34)	29 (25, 33)	32 (28, 35)
BMI (kg/m^2) Median (IQR)	27 (23, 31)	28 (23, 32)	26 (23, 31)
First pregnancy (n (%))	79 (32%)	24 (28%)	55 (35%)
Ethnic background (n (%))			
White British & European	89 (36%)	26 (30%)	63 (39%)
Pakistani	131 (53%)	49 (57%)	82 (51%)
Other	26 (11%)	11 (13%)	15 (9%)
Highest education level (n (%))[2]			
<5 GCSE or equivalent	32 (13%)	12 (14%)	20 (13%)
5+ GCSE or equivalent	58 (24%)	24 (28%)	34 (21%)
A-level equivalent	43 (17%)	11 (13%)	32 (20%)
Higher than A-level	105 (43%)	35 (41%)	70 (44%)
Other/ Don't know	8 (3%)	4 (5%)	4 (3%)
NS-SEC (n (%))[3]			
Managerial, administrative & professional	81 (33%)	22 (26%)	59 (37%)
Intermediate occupations or Small employers & own account workers	40 (16%)	14 (16%)	26 (16%)
Lower supervisory & technical or Semi-routine & routine	42 (17%)	16 (19%)	26 (16%)
Never worked	83 (34%)	34 (40%)	49 (31%)
Smoked in 1st trimester (n (%))	15 (6%)	5 (6%)	10 (6%)
Alcohol in 1st trimester (n (%))	17 (7%)	3 (3%)	14 (9%)
Vegan or vegetarian (n (%))	11 (4%)	<3	10 (6%)
Total iodide intake (diet & supplements) in pregnancy (μg/d) [1]			
Geometric mean (95% CI)	136 (126, 146)	97 (85, 111)	162 (150, 175)
Median (IQR)	143 (94, 196)	97 (76, 136)	168 (126, 226)
Total iodide intake in pregnancy compared to RNI (n (%)) [1]			
< WHO recommendations (250 μg/day)	219 (89%)	82 (95%)	137 (86%)
< UK RNI (140 μg/day)	119 (48%)	66 (77%)	53 (33%)
< UK LRNI (70 μg/day)	28 (11%)	19 (22%)	9 (6%)
Dietary iodide intake in pregnancy (μg/d) [1]			
Geometric Mean (95% CI)	96 (89, 105)	97 (85, 111)	96 (87, 106)
Median (IQR)	101 (64, 142)	97 (76, 136)	104 (62, 147)
Supplement use (n (%))			
Used any supplement prior to pregnancy [4]	80 (33%)	21 (24%)	59 (37%)
Used any supplement in 1st trimester	220 (89%)	64 (72%)	156 (98%)
Supplements containing iodide (n (%))			
Used supplement containing iodide prior to pregnancy [4]	26 (11%)	3 (3%)	23 (14%)
Used supplement containing iodide in 1st trimester	110 (45%)	0 (0%)	110 (69%)
Iodide intake from supplements in pregnancy (μg/d) [1]			
Geometric mean (95% CI)	12 (9, 16)	0 (0, 0)	54 (48, 60)
Median (IQR)	35 (0, 68)	0 (0, 0)	54 (36, 89)
Urinary iodine concentration in pregnancy (μg/l) [1]			
Geometric mean (95% CI)	131 (121, 143)	127 (112, 144)	134 (120, 150)
Median (IQR)	135 (90, 207)	126 (83, 200)	141 (90, 220)
Iodine:Creatinine in pregnancy (μg/g) [1]			
Geometric mean (95% CI)	128 (119, 137)	107 (95, 120)	140 (129, 153)
Median (IQR)	126 (88, 187)	109 (73, 138)	138 (95, 209)
Presence of any palpable goitre in pregnancy (n (%)) [1,5]	89 (36%)	25 (29%)	64 (40%)

Values are at baseline unless otherwise stated. Values are median (IQR) or n (%), unless otherwise stated

[1] Values are averages from data collected over 3 pregnancy visits.

[2] UK-equivalised overseas qualifications

[3] Some NS-SEC categories were combined because of small cell counts

[4] In the 3 months prior to pregnancy

[5] Grade 1 or 2 goitre, according to World Health Organisation 1994 criteria

Table S3: Dietary sources of iodide across all study visits, by ethnic group

	All n=246		Pakistani n=131		White British & European n=89		Other n=26	
	Geometric mean[2] (µg/d) (95% CI)	%[3]	Geometric mean[2] (µg/d) (95% CI)	%[3]	Geometric mean[2] (µg/d) (95% CI)	%[3]	Geometric mean[2] (µg/d) (95% CI)	%[3]
Total iodide intake (µg/d) Geometric mean (95% CI)	125 (118, 134)		117 (108, 127)		133 (121, 147)		143 (102, 199)	
Iodide intake from dietary sources (µg/d)	101 (94, 108)		96 (88, 105)		103 (94, 114)		119 (84, 169)	
Dietary iodide from each food source [1]								
Dairy	34 (30, 38)	41%	29 (25, 34)	37%	41 (33, 50)	46%	34 (22, 55)	39%
Dairy with cereal	7 (5, 8)	12%	5 (4, 6)	10%	10 (8, 12)	14%	6 (3, 10)	9%
Eggs	5 (4, 7)	13%	9 (7,12)	17%	3 (2, 4)	7%	2 (1, 4)	5%
Cereals	3 (2, 3)	4%	3 (2, 3)	4%	3 (2, 3)	4%	2 (1, 3)	5%
White Fish	2 (2, 3)	12%	3 (2, 4)	13%	1 (1, 2)	7%	7 (2, 20)	24%
Other seafood	1 (<1, 1)	2%	<1 (<1, <1)	2%	1 (<1, 1)	2%	1 (<1, 2)	3%
Poultry and red meat	7 (6, 7)	9%	7 (6, 8)	10%	6 (5, 7)	8%	6 (4, 9)	10%
Fruit, vegetables & pulses	4 (4, 5)	6%	4 (4, 4)	5%	5 (4, 6)	7%	4 (3, 5)	4%
Confectionery	1 (1, 1)	2%	<1 (<1, 1)	1%	2 (1, 2)	3%	1 (<1, 1)	1%
Other	<1 (<1, <1)	1%	<1 (<1, <1)	<1%	<1 (<1, 1)	1%	<1 (<1, <1)	<1%

[1] Category details are in Supplemental table 1

[2] Geometric means of individual dietary sources do not sum to geometric mean of total, because these are derived on the log scale to reflect skewed distribution of dietary intakes.

[3] Arithmetic mean percentage of each participant's diet from each food source.

Figure S1: Study participant flow chart

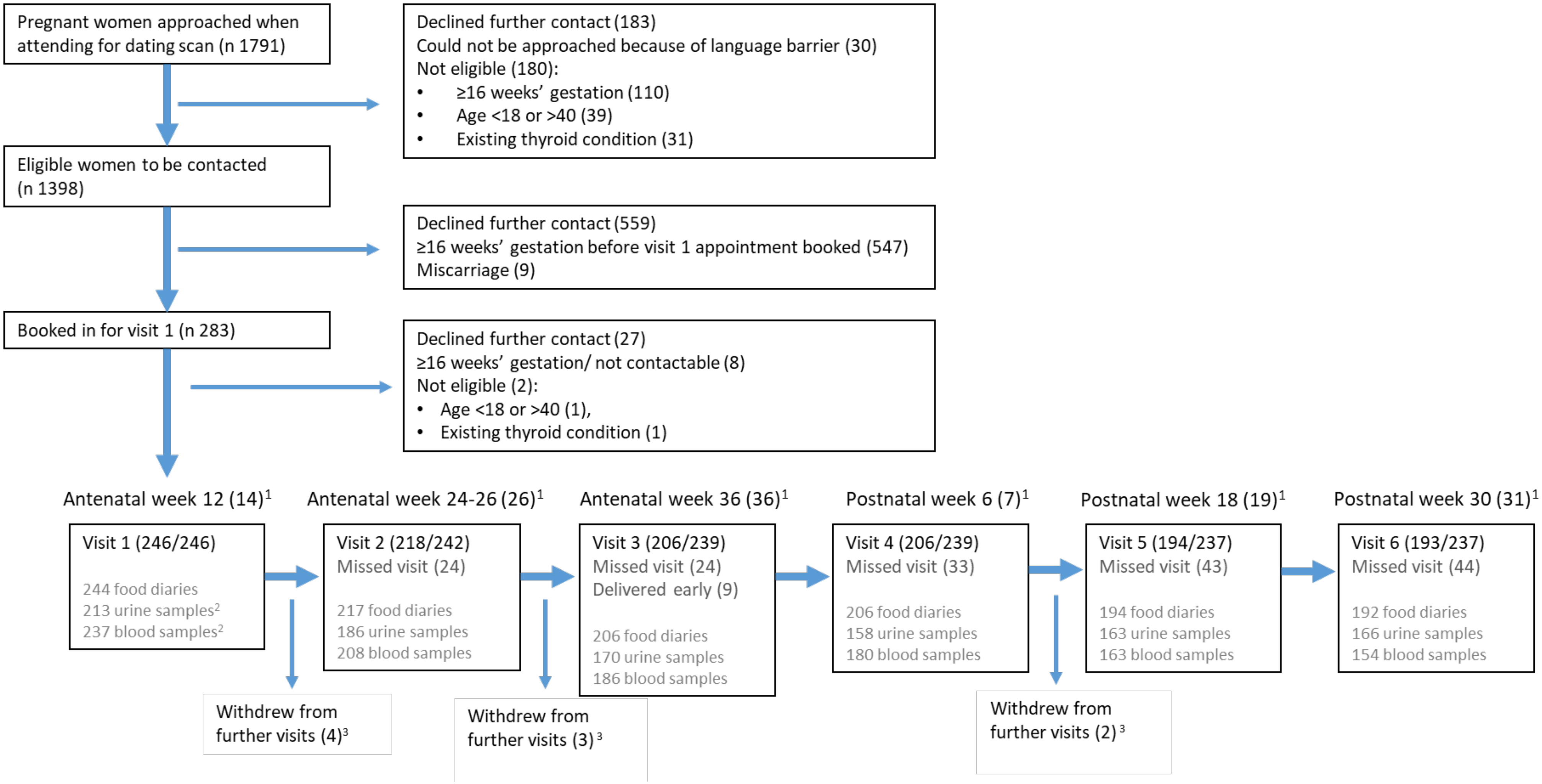

Notes:

[1]Target date (mean achieved date)

[2]Urine and blood sample numbers relate to viable samples with sufficient volume for testing

[3]Reasons for not participating in further visits include miscarriage, stillbirth, neonatal death, declined further contact and moved away. Numbers for each reason are withheld to avoid identifying individuals.
